# Applying the use of shared medical appointments (SMAs) to improve continuous glucose monitor (CGM) use, glycemic control, and quality of life in marginalized youth with type 1 diabetes: Study protocol for a pilot prospective cohort study

**DOI:** 10.1016/j.conctc.2023.101067

**Published:** 2023-01-16

**Authors:** Jody B. Grundman, Shideh Majidi, Amanda Perkins, Randi Streisand, Maureen Monaghan, Brynn E. Marks

**Affiliations:** a Division of Endocrinology, Children's National Hospital, 111 Michigan Ave NW, Washington, DC, USA; b George Washington University School of Medicine and Health Sciences, 2300 I St NW, Washington, DC, 20052, USA; c Children's Hospital of Philadelphia, 3401 Civic Center Blvd, Philadelphia, PA, 19104, USA

## Abstract

**Background:**

Continuous glucose monitors (CGMs) have been associated with improved glycemic control and diabetes-related quality of life in youth with type 1 diabetes (T1D), however use is lowest among youth from low-income households and racial/ethnic minorities. Shared medical appointments (SMAs) have been shown to improve glycemic control and reduce diabetes distress in adolescents with T1D, but a focus on marginalized youth has been lacking. This prospective cohort pilot study will assess feasibility and acceptability of the SMA intervention and impact on CGM uptake and sustained use, glycemic control, and diabetes distress in marginalized youth with elevated hemoglobin A1c (HbA1C).

**Methods:**

The pilot study will recruit 20 publicly insured youth with T1D aged 8–12 years who identify as non-Hispanic Black or Latinx and have had at least one HbA1C value > 8% in the past year and their primary caretaker. The trial will employ an enrollment visit, SMA visits every 3 months over a 12-month study period, and a 6-month follow-up observational period. Feasibility measures include proportion of eligible youth successfully recruited for participation, proportion initiating CGM, SMA attendance, and retention through study completion. Acceptability will be assessed using satisfaction surveys. Changes in glycemic control will be assessed using CGM metrics and A1c from baseline to completion of the 12-month SMA intervention, as well as 3 and 6-months after completion of the SMA intervention.

**Conclusion:**

Implementing SMAs for marginalized youth has the potential to address diabetes disparities by optimizing clinical and psychosocial outcomes for the most vulnerable youth living with T1D.

Trial Registration: https://clinicaltrials.gov/ct2/show/NCT05431686.

## 1 Introduction

Health disparities exist among individuals with type 1 diabetes (T1D) of different races, ethnicities, and socioeconomic statuses. Youth of color and those from the lowest socioeconomic statuses have worse long-term outcomes as compared to wealthier, non-Hispanic whites (NHW) in the U.S [1]. Minority youth with T1D are significantly more likely to have higher hemoglobin A1C (HbA1c) levels, elevated cardiovascular risk factors, and early signs of nephropathy, retinopathy, and neuropathy as compared to NHW youth [1]. In addition to differences in glycemic control and complications, it is also well established that youth of racial/ethnic minorities and of low socioeconomic status experience higher rates of diabetes distress [2,3].

Unfortunately, these disparities in outcomes mirror the reality of access to emerging diabetes technologies in clinical care and research, and Non-Hispanic Black (NHB) and Latinx youth are woefully underrepresented in diabetes technology trials. Continuous glucose monitor (CGM) use has been associated with lower HbA1c values, less hypoglycemia, and improved quality of life [[4], [5], [6]]. CGM has become the standard of care [7] for diabetes patients, and use has increased exponentially over the past decade due to improvements in accuracy, usability, and expanded access through insurance coverage [4,5]. Despite well established evidence associated with CGM use, NHB and Latinx youth are much less likely to use CGMs as part of T1D management, and worse, those who are not using these technologies report that they have not been offered the option to use these treatment modalities [8]. Using data from the T1D Exchange, Agarwal and colleagues reported that 72% of NHW young adults (ages 18–25) with T1D used CGM as compared to 18% of NHB young adults and 40% of Latinx young adults [9]. The drivers of health disparities in T1D technology access and use are complex and multifaceted.

Shared medical appointments (SMAs), wherein small groups of individuals participate in group education sessions in place of traditional clinic visits, have been successfully employed in pediatric chronic disease management, and are well suited for diabetes care [[10], [11], [12], [13], [14]]. SMA visits allow additional time for skill building, self-efficacy, peer support, and interprofessional self-management education with diabetes educators, nutritionists, physicians, and psychologists [15]. Specifically in pediatric T1D, SMAs have been shown to allow for discussion of a greater breadth of topics while also placing greater focus on behavioral and psychosocial needs [16,17]. Adolescents participating in SMAs report high satisfaction and exhibit improved glycemic control and reduced diabetes distress through the development of a supportive community [11]. Increased satisfaction with follow-up care can also translate into improved attendance and engagement in regularly scheduled clinical care. However, to date, the use of SMAs has not been well-studied in younger youth, or specifically in marginalized youth with T1D, and has not been used specifically to promote uptake and sustained use of diabetes technologies. Despite the well-known improvements in glycemic control with CGM use, the impact on diabetes distress in youth has been mixed [[18], [19], [20]]. Designing a clinical model that focuses on reducing diabetes distress and increasing CGM uptake in a cohort that has been shown to have high levels of diabetes distress and low levels of CGM use has the potential to minimize known barriers to optimal diabetes outcomes, and improve health equity among youth with T1D [21].

The primary aim of this pilot study is to assess the feasibility and acceptability of a 12-month SMA model employing interprofessional facilitator-mediated discussion to promote the uptake and sustained use of CGM, while also providing peer support, and fostering community to improve glycemic control and promote health equity among youth with T1D. Furthermore, this SMA intervention, focused on the needs of publicly insured NHB and Latinx youth with sub-optimally controlled T1D, will be the first specifically developed to support uptake and sustained use of diabetes technology. This SMA intervention will be designed specifically for younger youth, where the impact of SMAs has not been well studied. The SMA intervention is rooted in the tenets of social cognitive theory, which recognizes that knowledge, self-efficacy, and psychosocial factors all impact health behaviors [22]. We hypothesize that the SMA model will lead to high rates of SMA retention and satisfaction, greater CGM wear time and sustained use, improved glycemic control, and reduced diabetes distress compared to individuals in the traditional individual appointment model. The SMA intervention aims to improve uptake and sustained use of CGM, improved glycemic control, and reduced diabetes distress among youth who have historically had sub-optimal glycemic control, high rates of diabetes distress, and have been least likely to access CGM devices [[23], [24], [25]].

## 2 Methods

### 2.1 Study design

This pilot trial will utilize a prospective cohort design. The study will include a single intervention group participating in the SMA visits (n = 20). In addition to assessing acceptability and feasibility, a goal of this pilot trial is to obtain effect size estimates to power a larger scale randomized controlled trial. The trial is registered with the Clinical Trials Registry (NCT05431686). Study approval was obtained from the Children's National Hospital Institutional Review Board (Pro00016268). The study will be conducted at Children's National Hospital, a pediatric tertiary care center in Washington, DC.

### 2.2 Study eligibility criteria

The study inclusion criteria are: (1) aged 8–12 years, (2) T1D duration of at least 1 year, (3) at least one HbA1C value > 8% in the past year, (4) self-reported NHB race and/or Latinx ethnicity, (5) public health insurance, which is used as a proxy measure of socioeconomic status, (6) English fluency in the youth and parent, and (7) participation of the primary diabetes caregiver. English fluency is a requirement as we currently do not have the ability to run the SMA intervention in other languages. Enrollment is open to youth that are currently using CGM, as well as those who will commit to using CGM for the duration of the study period. The study exclusion criteria are: (1) Use of insulin pump therapy for diabetes management at time of enrolment, (2) major illnesses other than T1D, (3) significant cognitive limitations and major psychiatric disorders in the child or parent, (4) concurrent use of any non-insulin diabetes medication to control blood glucose levels, and (5) concurrent participation in any other clinical studies during study period.

### 2.3 Recruitment and enrollment

All youth scheduled for routine clinical diabetes appointments at Children's National Specialty Care Locations will be screened through chart review by study team members to assess for eligibility after a HIPAA authorization waiver for recruitment is granted. Study team members will review upcoming clinic lists for eligibility criteria, and eligible youth will be approached to participate during routine clinical visits. For youth who cannot be approached in person at the time of the visit or are still considering enrollment after the clinic appointment, we will attempt to contact primary caregivers by email, phone, and/or text message. Text messages will not contain PHI and provide general information about the project and how to contact the study team if interested**.** If an email address is not on file, a letter will be sent via mail.

Once a potential participant has been identified, a study team member will give an IRB approved study information letter to the participant's primary diabetes caregiver and legal guardian. If the caregiver expresses interest in the study, a copy of the IRB approved consent will be provided. A research team member will review the informed consent form with the family and explain the study and answer all questions prior to obtaining informed consent from the parent and assent from the child. The informed consent will be signed by the caregiver and assent will be obtained from the patient at an in-person visit before any research interventions are done.

For youth who cannot be approached in-person at the time of the visit, we will attempt to contact individuals by phone and obtain e-consent and/or e-assent with REDCap. The REDCap link of the IRB approved consent will be provided to the caregiver via text or email. A research team member will review the informed consent form with the family and explain the study and answer all questions prior to obtaining informed consent from the parent and assent from the child electronically. The informed consent will be electronically signed by the caregiver and assent will be obtained electronically from the patient.

### 2.4 Intervention

#### 2.4.1 SMA structure and follow-up

This trial will employ SMA visits every 3 months over a 12-month study period, and a follow-up 6-month observational period to continue to evaluate outcomes once youth return to routine individual follow-up care. SMAs will consist of 4–6 youth with T1D and their primary diabetes caregiver. The SMA visits will be in place of the routine diabetes clinic visits. A new SMA group will be formed when enough parent-child dyads have enrolled, and these groups will remain the same across the intervention. All youth and caretakers will complete standard check-in procedures with the clinic medical staff, including vital sign assessments, anthropometric measurements (weight, length, BMI, BMI Z-scores), and point-of-care fingerstick HbA1c measurements. CGM data from 14 days prior to each SMA will be reviewed by the endocrinologist ahead of the group visit, and parent-child dyads will meet with the endocrinologist one-on-one for up to 10 min to review individual diabetes management plans and any content that the youth or parent would prefer to discuss outside of the group setting.

During each visit, the facilitator role will be shared between a pediatric endocrinologist, certified diabetes care and education specialist (CDCES), nutritionist, and psychologist. Youth and caregivers will individually meet with the endocrinologist at different time points during the session to update personal management plans and goals of care. Each of the SMA sessions will be focused on one aspect of the content described in the International Society of Pediatric and Adolescent Diabetes (ISPAD) Clinical Practice Consensus Guidelines on delivery of ambulatory diabetes care and diabetes education [26,27] with a focus on issues that have been described in the literature as potential barriers to CGM use and adherence.

An overview of the 5 SMA sessions including content to be reviewed and key team members is outlined in Table 1. Before each session, dyads will be invited to submit questions to the research team that will then be anonymously reviewed and discussed among the group during the SMA. If no questions are submitted by families, the facilitator will begin the discussion by asking about goals that were set at the previous visit and experiences related to trying to reach these goals. The group visit will conclude with a review of the core concepts discussed. Youth will be encouraged to set and share individual goals to be addressed before the next SMA.

**Table 1:** SMA sessions content overview.

Session	Theme	Topics to Discuss	Key Team Members
1 (month 0)	Hypo/Hyperglycemia	Hypo/Hyperglycemia symptoms	Pediatric Endocrinologist CDCES
		Treating hypo/hyperglycemia	
		Hypoglycemia emergencies: glucagon	
		Hyperglycemia emergencies: ketones, DKA	
	CGM Background	How does CGM work	
		Parts of the CGM system	
		CGM insertion/removal	
		CGM alarms and alerts	
		CGM pros/cons	
2 (month 3)	CGM related distress	Distress/embarrassment with CGM alarms	Pediatric Endocrinologist CDCES
		Fear of hypoglycemia	Psychologist
		Stress related to hypoglycemia during exercise and sleep	
		Impact of CGM on family relationships	
	CGM report review	Interpreting CGM trend arrows	
		CGM report data: GMI, average glucose, TIR, TBR, TAR	
		Trend graph and daily reports: identify trends	
3 (month 6)	Nutrition	Review participants' current dietary habits	Pediatric Endocrinologist CDCES
		Healthy habits: food choices and portion sizes	Nutritionist
		Carb counting food labels, online nutrition resources	
		Sugar free/diabetic foods	
		Effects of certain foods on CGM trends	
	CGM report review	Interpreting CGM report data: GMI, average glucose, TIR, TBR, TAR	
		Trend graph and daily reports: identify trends	
4 (month 9)	Exercise and Sick Day management	Effects of exercise on blood glucose	Pediatric Endocrinologist CDCES
		Preventing hypoglycemia with exercise	
		Delayed hypoglycemia post-exertion	
		Sick day strategies: fever and hyperglycemia, vomiting/hypoglycemia, hyperglycemia with ketones	
	CGM report review	Interpreting CGM report data: GMI, average glucose, TIR, TBR, TAR	
		Trend graph and daily reports: identify trends	
5 (month 12)	Diabetes technology	Insulin pumps and hybrid closed loop systems	Pediatric Endocrinologist CDCES
		Review similarities and differences between different insulin pumps	Study Team Member unfamiliar to the group
		Brief overview of technology that is in development	
	Semi-structured focus group	How does SMA compare to traditional clinic visit experience	
		Impact of SMA on diabetes-related distress, quality of life, self-management skills, and glycemic control	
		Impact of SMA on attitudes and behaviors related to CGM use and CGM reports	

#### 2.4.2 Initial SMA (month 0)

The structure of the group visits will be reviewed with youth and families, and all members of the diabetes team will introduce themselves and explain their role in diabetes care. As some children will be CGM naïve before this visit, education will focus on insertion and removal of the CGM along with appropriate use of the CGM receiver, alerts and alarms, data sharing, and troubleshooting common CGM problems. Youth who are CGM naïve will be started on CGM technology at the initial SMA visit. Established sharing of CGM data between youth and the diabetes clinical care team by setting-up Dexcom Share and Follow applications will be confirmed before the end of the visit. The group visit will conclude with a review of the core concepts discussed, and youth and caregivers will set goals for the next SMA.

#### 2.4.3 Follow-up SMAs

Four follow-up SMAs will be scheduled 3, 6, 9, and 12 months from the initial group visit. SMA 1 will focus on recognition and management of hyper- and hypo-glycemia. During the session the facilitator will aim to address common challenges with CGM use related to hyper- and hypo-glycemia, including how recurrent alerts and alarms can result in alarm fatigue [18], and falsely low glucose readings and sensor or transmitter failures are common reasons for discontinuing CGM use [28]. SMA 2 will focus on diabetes related distress, and how the continuous nature of CGM data can create stress related to the constant need for diabetes-related attention [18,29]. Psychologists and CDCES will discuss strategies to cope with the on-body presence and potential embarrassment resulting from CGM wear and to optimize parent-child communication surrounding CGM. SMA 3 will focus on nutrition, and nutritionists will highlight the impact of different foods on glycemic trends and discuss strategies to optimize control. SMA 4 will cover sick day management and exercise, including how to adjust CGM alarms and interpret CGM data in the context of increased physical activity or acute illness. SMA 5 will review diabetes technology and the pros and cons of the various options available for youth with T1D.

SMAs will also allow ample opportunity to jointly review de-identified CGM reports and how to effectively interpret data and make management changes in response to CGM data, empowering children and families to improve self-management and reduce distress related to CGM use. Active participation in discussions and group exercises will continue to strengthen the community feeling and social support network within the SMA group. After the 12-month SMA, participants will return to standard clinical care with individual patient-provider visits.

#### 2.4.4 3-Month and 6-month post-intervention follow-ups

Survey measures and assessments of glycemic control will be repeated after 3 months and 6 months of routine clinical care to assess for long-lasting effects of the intervention. After the SMA visits have been completed, children will be scheduled for individual follow up visits with their primary endocrinologist 3-months and 6-months (±1 month) from the final SMA visit. Families will continue to be contacted ahead of their follow up visits to remind them to attend and the need to complete surveys at these visits.

### 2.5 Outcome measures

We will look at differences in the following measures from baseline to completion of the SMA intervention, as well as 3- and 6-months after completion of the SMA intervention. We expect to obtain very complete data, as parent-child dyads will receive ClinCard vouchers and parking passes provided they attend the SMA study visit and complete all required surveys. Any surveys that have not been completed ahead of the visit will be completed in-person at the time of the SMA visit.

#### 2.5.1 Primary outcome measures: feasibility and acceptability

Given the novelty of the SMA approach for marginalized youth and focus on CGM technology uptake and sustained use, feasibility and acceptability of the intervention will serve as the primary outcome measure. Feasibility is of great importance with a focus on youth from historically underrepresented racial and ethnic backgrounds and income levels. We will track feasibility using a CONSORT table that will include the proportion of eligible youth successfully recruited for participation, proportion initiating CGM, SMA session attendance rates, and rates of retention to study completion. Attrition data and team feedback will help determine how recruitment rates may be improved.

Acceptability will be assessed using focus groups and satisfaction surveys. The survey measures will include 5-point Likert scale items exploring participants’ satisfaction with participation, perceived utility of the intervention content, and perceived benefit from participation. During the final SMA parent-child dyads will participate in a semi-structured focus group to assess satisfaction with the SMAs. Focus groups will be led by a researcher not involved in the delivery of SMA visits to create an environment where respondents can feel comfortable being open and honest with their responses and minimize bias. Participants will be asked to share thoughts and opinions on the SMA model and how it compares to the routine individual clinic visit standard of care. To assess perceived benefits, participants will have the opportunity to speak freely about the impact that SMA has had on diabetes-related distress, quality of life, self-management skills, and glycemic control for both the caregiver and the child (if applicable). Participants will also be asked to assess how SMAs have influenced attitudes and behaviors related to CGM use and CGM reports.

#### 2.5.2 Secondary outcome measures: changes in glycemic control

CGMs will be prescribed for all participants, as they are covered by public health insurance. Locked smartphones will be supplied for the study duration so that CGM data can be shared. CGM metrics and chart review will be used to assess effects on glycemic control. (1) CGM time in range from 70 to 180 mg/dL (TIR), (2) time below range (<70 mg/dL) (TBR), (3) time above range (≥180 mg/dL) (TAR), (4) mean sensor glucose, (5) coefficient of variation (CV) of glucose, (6) CGM wear time, and (7) HbA1c. Glycemic indicators will be obtained from CGM downloads for the 14 days prior to follow-up data collection time point. As guided by published standardized CGM metrics [30], data will be transformed to identify number of measurements and percent TIR, time below range (<70 mg/dL) (TBR), and time above range (≥180 mg/dL) (TAR), mean sensor glucose, and coefficient of variation (CV) of glucose values during each 14-day period. We also will track the percent CGM wear time.

CGM time in range (TIR) from 70 to 180 mg/dL has been correlated with HbA1c values, which have classically been used as primary outcomes in T1D studies because of their association with microvascular complications [31]. We have chosen to focus on TIR rather than HbA1c because of the propensity for HbA1c variation in NHB youth who also have a higher propensity for hemoglobin variants [32]. Despite increased focus on TIR, HbA1c has been included because it has classically been considered the gold standard [33]. Additional CGM derived measures of glycemic control are commonly used outcomes in studies of T1D technology [34]. Any participant that chooses to discontinue CGM during the 12 month study period will be asked to wear a blinded CGM 10 days prior to the follow-up visits to allow for complete data collection.

#### 2.5.3 Tertiary outcome: changes in patient reported outcome measures

A battery of validated survey measures exploring diabetes-specific self-management skills and diabetes-related quality of life will be completed by the child and the primary diabetes caregiver at each of the SMA visits as well as 3- and 6-months post-intervention, including: Type 1 Diabetes and Life (T1DAL) [35], Diabetes Self- Management Profile (DSMP) [36], Self-Efficacy for Diabetes (SEDS) [37], and Problem Areas in Diabetes- Child and -Parent (PAID-C; *P*-PAID-C) [38]. Perceived benefits and barriers to CGM use will be assessed using the CGM Benefits and Burdens scale [39]. Surveys will be completed by a single, consistent parent/caregiver throughout the study, preferably the one most involved in medical care.

### 2.6 Data analysis plan

Descriptive measures will be used to summarize participant demographic information.

*P*-values and confidence intervals will be two-tailed. Covariates will be pre-specified. Tests to assess normality will be performed and data transformation or non-parametric analyses will be used when necessary.

#### 2.6.1 Analysis of the primary Endpoint(s)

Multiple studies evaluating redesigning clinical care models in adult patients with type 1 and type 2 diabetes have used improvements in process measures, glycemic control, knowledge, and quality of life to assess for feasibility, and session attendance to assess acceptability [[40], [41], [42], [43]]. We will assess feasibility by tracking recruitment, enrollment, participation, and retention through a CONSORT table striving for benchmarks for each item as detailed in Table 2. Feasibility benchmarks include the following targets: recruitment of >60% of reached/eligible participants, >80% CGM initiation, >80% SMA sessions attendance, and >80% retention throughout the study.

**Table 2:** Analytical plan for feasibility and acceptability.

Construct	Measure	Benchmark Goals	Interval
Recruitment	# referred		Study initiation through completion of recruitment
Feasibility/	# screened		
Acceptability	# enrolled		
	% eligible who enroll	>60% reached/eligible youth enrolled	
Intervention	#/% sessions attended	>80% session attendance	SMA visits every 3mo
Feasibility			
Intervention	Child/parent satisfaction ratings	>80% report satisfaction	After completion of intervention
Acceptability	Child/parent qualitative interview	>80% reporting perceived utility	
Retention	% who initiate CGM	>80% CGM initiation	All assessment time points
Feasibility/Acceptability	% who continue on CGM	>80% continue to wear CGM	
	% completing follow-up assessments	>80% complete follow up assessments	
Assessment	% participants with missing HbA1c data	<10% with missing HbA1c data	All assessment time points
Feasibility	% participants with missing CGM data	<10% with missing CGM data	
	% participants with missing survey data	<10% with missing survey data	

Acceptability will be assessed using satisfaction surveys and determined by a high level of satisfaction with participation, perceived utility of the intervention content, and perceived benefit from participation. Acceptability measures consist of >80% reporting satisfaction and >80% reporting perceived utility on user satisfaction ratings.

We will report summary statistics on each feasibility and acceptability item in the questionnaires (mean and standard deviation well as percent answering a specific Likert level).

Thematic analysis of qualitative data obtained from the focus groups will be conducted by two team members who will independently review focus group transcripts to generate initial codes. Initial codes will be discussed by the group to generate a list of second-cycle codes and each team member will then apply the coding framework to all transcripts before identifying dominant themes. NVivo software will be used to organize and analyze the qualitative data.

#### 2.6.2 Analysis of the secondary Endpoint(s)

CGM data will be analyzed using library GLU in R using mixed effect models. These models will quantify the impact of our intervention on glucose control over time. The independent variables will be baseline time in range, child age, and a random subject effect to account for correlation of measurements on the same subject. Interaction terms of age and baseline time in range with the intervention will explore possible effect modification (p < 0.10) of these characteristics. We will also explore differences between time points and average outcome by parent income. Results will be reported as least-squares means (LSMEANS) with standard errors. Casewise deletion will be used to handle missing data.

#### 2.6.3 Analysis of the tertiary Endpoint(s)

We will summarize self-management skills and diabetes-related quality of life by different time points. Differences in participant self-management skills and patient satisfaction will be reported as mean Likert scale score and standard deviation. Similar analysis using linear longitudinal regression analysis will be conducted. Results will be reported as least-squares means (LSMEANS) with standard errors. Casewise deletion will be used to handle missing data.

#### 2.6.4 Sub-group analyses

The primary and secondary endpoints will be analyzed without regard to age and sex given the narrow inclusion criteria for age and the lack of evidence to suggest sex-based differences in glycemic control among youth with T1D. Race/ethnicity will not be factored in as the study will not have a large enough sample size and sufficient power to stratify findings based on race/ethnicity.

#### 2.6.5 Sample size

As this is a pilot study, we plan to enroll up to 20 parent-child dyads. We anticipate low dropout rates, but do plan to enroll new subjects to keep the total number of subjects at up to 20 in case of attrition. If there is a sufficient number of new subjects enrolled to replace those that drop out, a new SMA group will be created so that newly enrolled subjects will be able to start the intervention from the baseline visit.

## 3 Discussion

We expect that the SMA model will lead to high feasibility and acceptability scores, improved CGM uptake and sustained use, improved glycemic control, and improved diabetes-related quality of life. The American Diabetes Association (ADA) recommends that education and social support is continually offered to youth and their caregivers at routine clinic visits [44], however this is challenging within the constraints of the current clinical care model. Specifically in pediatric T1D, SMAs have been shown to allow for discussion of a greater breadth of topics while also placing greater focus on behavioral and psychosocial needs [16,17]. Low-income and racial/ethnic minority youth with T1D are less likely to have regular outpatient diabetes follow-up care [45], and report additional difficulties with accessing quality diabetes care, such as culturally appropriate education [46]. The interprofessional SMA model incorporating CDCES, psychology, and nutrition support that is specifically designed for marginalized youth will allow for sufficient time to focus on culturally-competent education and increase access to diabetes technology, and result in improved adherence and health outcomes. These SMA visits will be in place of the routine individual clinic visits and comparable in duration of time spent in the clinic, so there will not be an added burden on patients participating in the study intervention.

Limitations of this pilot study include the small sample size, which might be under-powered to detect significant changes in measures of glycemic control. Although the sample size is small, we do anticipate that it is sufficient to evaluate the primary outcome of feasibility and acceptability. The inability to conduct the SMAs in languages other than English does narrow the eligibility criteria and limits participation of ethnic minorities. If the SMA model is confirmed to be feasible and acceptable among marginalized youth with T1D, we plan to recruit staff that are fluent in other languages for future studies so that English fluency will not be required for participation. Although SMAs In T1D have been studied in adults and adolescents, we believe that designing content that is culturally sensitive, developmentally appropriate, and tailored to a younger demographic will be valuable in assessing if this model is feasible in a younger marginalized cohort.

Diabetes-specific emotional distress and quality of life are psychosocial variables that are strongly associated with key diabetes outcomes, such as adherence and glycemic control [47,48]. Having diabetes requires many behavior changes, which can lead to stress in both the child and their families [49]. There has been very little described in the literature regarding diabetes-distress in marginalized or low socioeconomic status youth. Butler et al. demonstrated that NHB and Latinx young adults with T1D have increased diabetes-related distress that was associated with out-of-range glycemic control in Latinx compared to NHW young adults [2]. Similarly, data from the SEARCH for Diabetes in Youth study found quality of life was lowest among young adults with T1D in low-income marginalized youth [50]. Adolescents who participated in SMAs were found to experience reduced diabetes distress through the development of a s supportive community [11]. We anticipate that SMAs specifically designed for marginalized youth of a younger age group will provide youth and families with strategies to cope with the psychosocial burdens and challenges needed to optimize adherence and outcomes.

Lessons learned from these comprehensive interprofessional SMAs will also provide insights that will allow for development of additional strategies needed to promote equal opportunity for all youth to achieve health and quality of life targets without being hindered by race, ethnicity, or socioeconomic status. Findings from this pilot study will serve as the foundation to conduct larger-scale studies evaluating the implementation and application of SMAs for marginalized youth with T1D. We aim to utilize data on the feasibility and acceptability of this pilot SMA intervention to revise the educational content and design an SMA program that can be implemented on a larger scale to address the needs of diverse youth and their families that have not been adequately addressed in the current individual visit model, with the goal of improving health equity.

## Funding

This work was supported by the 10.13039/100000041American Diabetes Association [grant number #7-21-PDFHD-09]; and Investigator Initiated Research funding from 10.13039/100015769Dexcom Inc.

## Declaration of competing interest

The authors declare that they have no known competing financial interests or personal relationships that could have appeared to influence the work reported in this paper.
